# Greening vision: balancing clinical excellence and ecological sustainability in eye health

**DOI:** 10.3389/fpubh.2025.1685240

**Published:** 2025-10-10

**Authors:** Bin Lin, Jing Tang, Wei Liang, Peng Shi, Dong-kan Li

**Affiliations:** ^1^Xiamen Eye Center and Eye Institute of Xiamen University, Xiamen, China; ^2^Xiamen Clinical Research Center for Eye Diseases, Xiamen, Fujian, China; ^3^Xiamen Key Laboratory of Ophthalmology, Xiamen, Fujian, China; ^4^Fujian Key Laboratory of Corneal and Ocular Surface Diseases, Xiamen, Fujian, China; ^5^Xiamen Key Laboratory of Corneal and Ocular Surface Diseases, Xiamen, Fujian, China; ^6^Translational Medicine Institute of Xiamen Eye Center of Xiamen University, Xiamen, Fujian, China; ^7^Xiamen Humanity Rehabilitation Hospital, Xiamen, China

**Keywords:** ophthalmic sustainability, carbon footprint, green ophthalmology, environmental paradox, sustainable healthcare

## Abstract

The rapid advancement of ophthalmic medicine has significantly improved global visual health but concurrently imposed substantial ecological costs, creating an environmental paradox between efficient treatment and sustainability. This review explores the multifaceted carbon footprint of ophthalmic practices through a three-dimensional analysis: spatially, revealing stark cross-national differences in surgical emissions; temporally, tracking the environmental impact of technological evolution from extracapsular cataract extraction to phacoemulsification and vitreous surgery; and technologically, highlighting the role of disposable instruments, biomaterials, and energy consumption. It further presents an innovation matrix for “green ophthalmology,” encompassing technological breakthroughs, process optimizations, and behavioral interventions. The review emphasizes the need to integrate the “triple bottom line” (clinical, economic, environmental) into practice and policy, proposing future directions such as blockchain-based certification systems and standardized environmental assessment tools. Ultimately, it calls for multi-level actions-from individual clinicians to global governance-to reconcile high-quality eye care with ecological sustainability.

## 1 The environmental paradox in ophthalmic healthcare: the duality of efficient treatment and ecological costs

The rapid advancement of ophthalmic medicine has improved global visual health, but it has also incurred significant ecological costs, attributable to multiple factors. Take cataract surgery as an example: literature indicates that the greenhouse gas emissions from phacoemulsification cataract surgeries performed in UK hospitals are over 20 times higher than those of the same procedure in India (1). This discrepancy underscores the potential excessive environmental burden incurred by high-income countries in their pursuit of healthcare safety standards. Beyond the impacts of cataract surgery, the widespread adoption of anti-VEGF therapies, while addressing blinding conditions such as diabetic retinopathy, has also increased the carbon footprint due to issues like poor patient compliance (2), the short half-life of the drugs (3), and the requirements for cold chain transportation. As one of the most frequently accessed specialties within the healthcare system, the expansion of ophthalmic services-particularly in the Asia-Pacific region-necessitates a re-evaluation of the balance between “efficiency” and “sustainability.” The case of Indian hospitals has proven that process optimization can achieve a win-win situation for both clinical efficacy and ecological benefits.

A vicious cycle exists between climate change and eye health: on the one hand, ophthalmic healthcare activities contribute 8.5% of the total greenhouse gas emissions from the global healthcare system (4); on the other hand, global warming threatens visual health through multiple pathways. Enhanced ultraviolet radiation accelerates the development of pterygium and cataracts, while air pollution (such as PM2.5) is positively correlated with age-related macular degeneration (5, 6). Studies have pointed out that environmental degradation exacerbates climate vulnerability in fragile regions like Somalia (7), a pattern that also applies to ophthalmic diseases in low-income countries. When responding to outbreaks of climate-related eye diseases, responders are often forced to adopt emergency medical solutions with high environmental costs.

From the interdisciplinary perspective of environmental medicine, it is imperative to establish standardized assessment systems (such as life cycle assessment) to measure the full-chain ecological impacts of ophthalmic interventions. Simultaneously, the development of climate-adaptive treatment strategies is necessary to break this negative feedback loop (8, 9).

## 2 Three-dimensional analysis of ophthalmic carbon footprint

### 2.1 Spatial dimension: cross-national comparative studies

Based on existing literature analysis, there are significant cross-national differences in the carbon footprint of ophthalmic surgeries. Studies have shown that the amount of waste generated per surgery in India’s Aravind Eye Care System is only 0.504 kg, which is far lower than the 2–3 kg per surgery in Western countries (10). This discrepancy mainly stems from three factors: (1) Divergent strategies in the use of high-value consumables, with India adopting centralized procurement and strict cost control; (2) Differences in anesthesia methods, as developing countries rely more on local anesthesia; (3) Equipment recycling mechanisms, where the Aravind system achieves optimal resource allocation through large-scale surgeries (over 50 cases per day on average).

As previously mentioned, the carbon emissions from cataract surgeries in UK hospitals are over 20 times higher than those in their Indian counterparts, primarily due to differences in energy structures and patient transportation-related emissions—India utilizes more renewable energy sources (1). In U. S. operating rooms, carbon emission hotspots are concentrated in the production of disposable consumables (accounting for 27–43%) and inhaled anesthetic gasses (11, 12). In contrast, India has reduced environmental impact through innovative practices such as standardized instrument disinfection and customized surgical kits (13). These findings suggest that the intensive operational models of low-income countries may provide emission reduction insights for high-income countries, though adjustments must be made by localized medical regulations (4).

### 2.2 Temporal dimension: history of technological evolution

The technological evolution from extracapsular cataract extraction (ECCE) to phacoemulsification has led to a significant increase in environmental costs, primarily driven by the high energy consumption resulting from the continuous upgrading and iteration of operating room equipment in high-income countries (14), accompanied by the use of disposable consumables and anesthetic gasses. In vitreous surgery, the application of long-acting gasses (such as SF6 and C3F8) has improved the success rate of retinal reattachment; however, their global warming potential (GWP) is 23,900 times that of CO₂ (in the case of SF6) (4), creating a contradiction between therapeutic breakthroughs and environmental costs.

Temporal dimension analysis shows that the carbon footprint of phacoemulsification is mainly concentrated in the intraoperative phase (approximately 100–241 kg CO₂e per case) (11, 15), with biomedical waste and energy consumption being the dominant contributors. The carbon footprint of vitreous surgery, on the other hand, must account for the long-term impact of gas emissions-for instance, SF6 persists in the atmosphere for up to 3,200 years. Current research calls for balancing clinical efficacy and environmental sustainability through measures such as optimizing the recycling of consumables, improving gas recovery technologies (e.g., replacing SF6 with C2F6 can reduce GWP by 68%) (16), and establishing regionalized surgical centers to reduce transportation emissions.

### 2.3 Technical dimension

The use of disposable ophthalmic instruments significantly increases the surgical carbon footprint. Studies have shown that the carbon footprint of a single surgery ranges from 6 to 814 kg CO₂e, with 23% of instrument types contributing 80% of the emissions (such as single-use surgical drapes, surgical gowns, etc.) (15). The environmental sustainability of biomaterials needs to balance the degradation cycle with intraocular application requirements: although electrospun intelligent biomaterials have degradability and antibacterial properties (17), single-use products such as contact lenses still pose a high pollution risk, with annual waste in the United States reaching 2.8 billion pieces (18) Reusable processed instruments can reduce the carbon footprint by 50–67% (19), but issues such as biocompatibility of intraocular implants and residual sterilization need to be addressed (20, 21). Optimization from the technical dimension should integrate life cycle assessment (LCA), focusing on energy efficiency and material innovation (22).

## 3 Innovation matrix for green ophthalmology

### 3.1 Technological innovation

The innovative application of intelligent drug delivery systems in the field of ophthalmology has significantly improved treatment accuracy and reduced drug waste (23). Studies have shown that biodegradable drug carriers (LDBCs) responsive to the tumor microenvironment can achieve efficient combination therapy, with their pH-responsive properties increasing the drug release rate in target tissues to 70% (only 15% in a neutral environment) (24, 25). Meanwhile, biodegradable hydrogels developed using photopolymerization 3D printing technology can realize localized drug-controlled release, and such materials have been successfully applied in the treatment of retinal and optic nerve injuries (9, 26).

Ophthalmic intelligent delivery systems are moving toward nanotechnology, including cutting-edge fields such as gene delivery, cell therapy, and retinal implant devices (27, 28). In terms of carbon-neutral operating room design, 8.5% of greenhouse gas emissions from the U.S. healthcare system come from ophthalmic surgeries (4), making the research and development of biodegradable surgical materials a key priority. Biodegradable polymers are not only used in sutures and implants but can also serve as platforms for the sustained release of therapeutic agents (29).

Virtual reality surgical training systems reduce resource consumption in actual surgeries through 3D stereoscopic vision and haptic feedback (30). In addition, the intelligent food packaging technology using natural carbon dots has inspired green innovation paths for operating room consumables (31). Together, these technologies form a comprehensive solution for carbon neutrality in ophthalmic surgeries (32).

### 3.2 Improvement of treatment processes

In terms of process reengineering for green ophthalmology, resource integration in day surgery centers significantly reduces carbon emissions by optimizing equipment utilization and reducing energy consumption. Previous studies have highlighted the environmental cost issues of surgical processes in high-income countries (1). The surgical carbon footprint can be reduced by adopting reusable surgical instruments, improving operating room energy efficiency, and optimizing processes (12).

In addition, the time–space optimization strategy for anti-VEGF therapy can enhance sustainability by reducing the frequency of patient visits and drug waste. Since anti-VEGF biosimilars entered the U.S. market in 2022, their standardized production processes and optimized cold chain transportation have further reduced the treatment-related environmental burden (33).

Tele-ophthalmology makes a significant contribution to reducing carbon emissions by decreasing patients’ transportation needs. A systematic evaluation shows that telemedicine can reduce patient travel-related carbon footprints, especially in low-resource areas. AI-assisted remote screening (such as community-based fundus disease screening) can achieve dual optimization of environmental and cost benefits (34, 35). The tele-ophthalmology model supported by digital technologies (such as 5G and the Internet of Things) not only reduces transportation emissions by 90% but also improves service accessibility through process reengineering (36).

### 3.3 Behavioral interventions

In the field of green ophthalmology, the Nudge theory can enhance the environmental protection behaviors of medical staff through intervention strategies such as environmental cues, default options, and social norms (37). Studies have shown that environmental responsibility training based on social identity theory can significantly strengthen the green innovation behaviors of medical staff (38), while dynamic norm intervention has a significant effect on raising awareness of reducing plastic waste (but attention should be paid to possible reverse effects) (39, 40).

For patient education, the nudge strategy combined with self-determination theory (SDT) can increase the predictive validity of environmental attitudes on behavioral intentions by 81% (41), but it is necessary to pay attention to the cost threshold effect of behavior change-only low-cost behaviors are easy to change (39). The Nudge design in electronic health records can simultaneously improve clinical decision-making and ecological practices (42).

## 4 Sustainable development balancing act: the triple bottom line of clinical, economic, and environmental factors

Studies have shown that integrating the “triple bottom line” framework (economic, environmental, and social sustainability) into healthcare quality improvement is crucial (43, 44).

- In terms of infection control thresholds, in scenarios involving the reuse of instruments, it is necessary to evaluate infection prevention measures using five economic analysis methods (cost-effectiveness, cost-utility, etc.) (45), among which the incremental cost-effectiveness ratio (ICER) is a key indicator for assessing intervention measures (46). Research suggests adopting a risk-based stratified implementation strategy rather than a universally uniform approach (47).
- Cost–benefit analysis indicates that sustainable healthcare measures need to balance initial investment and long-term returns. Innovative methods such as membrane technology can improve both efficiency and cost-effectiveness by optimizing processes (48), while biochar catalysts play an important role in environmental protection due to their cost-effectiveness and multifunctional properties (49). Cost-utility analysis of digital healthcare strategies shows that certain interventions can achieve cost-effectiveness advantages after price adjustments (50).
- At the policy and regulatory level, establishing a standardized sustainable healthcare evaluation system is an urgent task (51, 52). Research recommends drawing on the “5R” principle (Refuse, Reduce, Reuse, Repurpose, Recycle) to formulate operating room emission reduction policies (53), while it is necessary to improve the regulatory framework to coordinate the dimensions of sustainable development (54). The hypercyclic healthcare model needs to integrate elements such as green leadership and green finance (55) and achieve dual improvement in environmental and cost-effectiveness by optimizing manufacturing processes (56).

## 5 Future vision of ophthalmic environmental protection

Previous studies have shown that patients undergoing ophthalmic surgery are more likely to seek medical treatment across different regions, which is consistent with the phenomenon we have observed in clinical practice (57). When seeking medical care across regions, patients may face situations where inspection results cannot be mutually recognized between different hospitals and medical records cannot be synchronized, leading to repeated examinations. Blockchain technology can specifically address this issue. Essentially, blockchain technology is a decentralized distributed ledger technology, characterized by core features of “immutable, traceable, transparent, and openly verifiable” data. Data is serially linked in chronological order in the form of “blocks,” and each node (e.g., hospitals, medical consumable manufacturers, regulatory authorities) can participate in data recording and verification without relying on a single central institution. This enables it to effectively address the challenges of data trust and traceability (58, 59). This technology is conducive to solving the current pain points in green ophthalmology practice, such as opaque data, difficult traceability, and inconsistent standards, while avoiding risks associated with unrecognized inspection data and waste caused by repeated examinations.

In terms of carbon trading, blockchain with PoS consensus mechanisms, such as Ethereum, can reduce carbon footprints by 99% (60), providing technical support for building an ophthalmic carbon trading market. Existing studies have pointed out that climate-smart medical measures such as optimizing supply chains, promoting telemedicine, and adopting bioaffinity designs can significantly reduce carbon emissions (61, 62).

In the future, it is necessary to develop standardized environmental assessment tools, combine blockchain with the Internet of Things, and build a closed-loop system covering consumables traceability, energy management, and carbon asset trading (63, 64), ultimately realizing the quantifiable and traceable environmental benefits of ophthalmic diagnosis and treatment.

## 6 Action initiatives: from individual practice to global governance

Promoting environmental sustainability in the field of ophthalmology requires multi-level actions—spanning individual clinicians, professional bodies, regional institutions, and transnational alliances—to translate theoretical insights into tangible practice. Clinicians can lay the groundwork by using standardized tools for measuring environmental impacts (1), which help quantify carbon emissions from daily procedures and identify reduction opportunities. Professional associations, meanwhile, should establish unified environmental performance indicators (65) and promote the spatial spillover effects of green financial policies (66) —providing frameworks and incentives for institutions to adopt sustainable practices, as exemplified by China’s localized reforms and global adaptive models below.

### 6.1 Practical lessons from the Chinese context

The Chinese ophthalmic community has developed hybrid sustainability models by selectively integrating Western technologies with Indian efficiency principles. At our center, we recognized the need to align ophthalmic development with low-carbon environmental protection in 2020 and thus initiated reforms by adopting targeted measures: we established a recycling system while upholding EU-grade sterilization protocols and integrated it into our daily clinical operations. Concurrently, we participated in building provincial tele-ophthalmology networks to expand access to eye care in rural areas. According to our 2024 annual statistics, these reforms have yielded tangible results—compared to 2020, we have reduced surgical waste by 34% without compromising safety, and the tele-ophthalmology networks now covering over 10 million rural residents have cut patient travel emissions by 26%. These experiences demonstrate that green ophthalmology is operationally feasible when combining the quality standards of high-income countries with the circular economy approaches of low-resource settings.

Transnational industrial alliances need to reconcile the contradictions between environmental goals and ophthalmic operational costs (67), facilitate inter-enterprise cooperation through big data technologies (68), and address specific issues such as drug redistribution (69).

### 6.2 Implementation roadmap for global practitioners

For health systems seeking immediate action, we recommend three priority steps: (1) Establish mutual recognition systems for diagnostic results among regional medical institutions to eliminate redundant testing; (2) Transition from disposable to reusable surgical textiles where clinically appropriate, and rationalize the use of pre-packaged pharmaceuticals and implants based on actual clinical needs to reduce unnecessary production and waste; (3) Develop regional equipment sharing platforms (e.g., for costly ophthalmic diagnostic or surgical equipment) inspired by successful international models but tailored to local regulatory frameworks, utilizing smart management systems for optimal scheduling and maintenance to maximize utilization rates. This phased approach strikes a balance between immediate improvements and long-term systemic transformation.
